# Isolation and pathogenicity of the novel porcine reproductive and respiratory syndrome virus 1 strain ZJ01 in South China

**DOI:** 10.3389/fcimb.2025.1646425

**Published:** 2025-07-25

**Authors:** Bohua Ren, Ruying Liu, Feibao Huang, Yu Wu, Xiaopeng Gao, Haishen Zhao, Limiao Lin, Qunhui Li, Xiangbin Zhang

**Affiliations:** ^1^ College of Animal Science, South China Agricultural University & Guangdong Provincial Key Lab of Agro-Animal Genomics and Molecular Breeding, Guangzhou, China; ^2^ Wen’s Group Academy, Wen’s Foodstuffs Group Co., Ltd., Xinxing, Guangdong, China; ^3^ Lingnan Modern Agricultural Science and Technology Guangdong Provincial Laboratory Yunfu Sub-center, Yunfu, China

**Keywords:** PRRSV-1, whole genome sequencing, evolutionary genetics analysis, GP3/4, phylogenetic analysis

## Abstract

**Introduction:**

The emergence and widespread dissemination of novel Porcine Reproductive and Respiratory Syndrome Virus type 1 (PRRSV-1) strains in China pose significant challenges, leading to substantial clinical infections within swine populations.

**Methods:**

In this study, we isolated a novel PRRSV-1 strain, designated ZJ01.

**Results:**

Whole-genome sequencing revealed a genome length of 15,125 bp. Comprehensive phylogenetic analysis based on the complete genome sequence classified ZJ01 within a distinct new sublineage. Intriguingly, phylogenetic analysis of the ORF5 gene indicated that ZJ01 clusters with classical BJEU06-1-like strains. Molecular characterization identified unique deletions within the hypervariable regions of structural proteins: a three-amino acid deletion at positions 243-248 in GP3 and a four-amino acid deletion at positions 63-68 in GP4. Pathogenicity studies in piglets demonstrated that ZJ01 infection induces characteristic clinical signs, including pyrexia and sustained viral shedding. Post-mortem examination revealed significant lung pathology characterized by hemorrhages and lesions typical of PRRSV infection. Notably, while causing significant morbidity, the ZJ01 strain did not result in direct mortality in the infected piglets under the conditions of this study.

**Discussion:**

Collectively, this study provides a detailed characterization and pathogenic evaluation of the novel PRRSV-1 strain ZJ01, contributing essential information for the development of effective prevention and control strategies in swine farms.

## 1 Introduction

Porcine reproductive and respiratory syndrome (PRRS) is a highly contagious disease caused by Porcine reproductive and respiratory syndrome virus (PRRSV) (Meulenberg, 2000; Wu et al., 2024). PRRSV is characterized by severe reproductive failure in sows manifesting as late-term abortions, stillbirths, mummified fetuses, and weak-born piglets and acute respiratory disease with high mortality rates in neonates and septicemia in affected herds (Dokland, 2010; Stadejek et al., 2013; Lunney et al., 2016).

The etiological agent was first recognized following major outbreaks in North America in 1987 (Dewey et al., 2000). Shortly thereafter, the prototype European strain, PRRSV-1, was isolated in 1991 from a pandemic affecting swine herds across Western Europe (Wensvoort et al., 1991; Ropp et al., 2004). This seminal isolate, designated Lelystad virus (LV), became the reference strain for PRRSV-1. Although LV initiated the pan-European pandemic and remains a critical benchmark, subsequent extensive genetic analyses revealed that it represents only a single lineage and does not reflect the full genetic diversity of contemporary PRRSV-1 field strains circulating globally (Meulenberg et al., 1995).

Based primarily on sequence divergence within the open reading frame 5 (ORF5) gene, which encodes the major envelope glycoprotein (GP5) (Firth et al., 2011), PRRSV-1 isolates are currently classified into distinct subtypes. Subtype I has demonstrated significant transcontinental spread, becoming established in Asia and the Americas (Gao et al., 2017). In contrast, other recognized subtypes (Subtypes II, III, and IV) remain predominantly confined to European swine populations, exhibiting more restricted geographical distributions. This subtype classification underscores the complex evolutionary dynamics and heterogeneous nature of PRRSV-1 (Gao et al., 2017).

Since the PRRSV-1 strain was first identified in China in 1997 (Meulenberg et al., 1997a), the virus has spread in nearly 20 provinces in China and differentiated into four major clades (Zhao et al., 2021). After 2006, the emergence and persistence of the BJEU06-1-like strain attracted more attention (Wang et al., 2016). Surveillance in 2018–2020 showed that PRRSV-1 was found in breeding farms, slaughterhouses, and clinical samples, and was often co-infected with PCV2 resulting in high mortality (Sun et al., 2023). The GZ11-G1 strain isolated in 2011 is highly homologous to the vaccine strain Amervac and may be related to vaccinated breeding pigs. In recent years, the number of tests for PRRSV-1 strains in China has increased rapidly (Wang et al., 2023).

Recently, there has been a significant increase in the detection rate of PRRSV-1 in clinical samples, which has had a significant impact on pig breeding results. To this end, we carried out PRRSV1 isolation, whole genome sequencing and pathogenic model study of piglets, with the aim of monitoring and studying the current prevalence and mutation of PRRSV1 strains in China.

## 2 Materials and methods

### 2.1 Sampling and virus isolation

Clinical tissue samples were triturated with antibiotic-containing DMEM medium, subsequently stored at -80°C, and three freeze-thaw processes were performed. Next, centrifuge the milled mixture and remove the supernatant. Total RNA was isolated from the supernatant using the RNeasy kit (Magen, China). PRRSV was detected by RT-PCR. Samples that test positive are diluted with culture medium, filtered through a 0.22 micron membrane, and seeded into primary alveolar macrophages (PAMs) for virus isolation.

### 2.2 RT-PCR amplification and sequencing analysis

Total RNA was isolated from cell culture samples using the RNeasy Kit (Qiagen, Hilden, Germany), following the manufacturer’s protocol. Viral genomic fragments were amplified using primers derived from published PRRSV sequences, maintained within our laboratory repository. Complementary DNA (cDNA) synthesis was performed using the PrimeScript™ RT-PCR Kit (Promega, Madison, WI, USA). Subsequent PCR amplification employed PrimeSTAR^®^ GXL DNA polymerase (TaKaRa, Shiga, Japan). The resulting amplicons were cloned into the pMD19-T vector (TaKaRa) via the TOPO^®^ TA Cloning^®^ Kit (Invitrogen, Carlsbad, CA, USA). Cloned products were commercially sequenced by Sangon Biotech (Shanghai, China) using the Sanger method. To ensure accurate sequence determination of the target region, a minimum of three independent clones were sequenced for each amplicon.

### 2.3 Immunofluorescence assay

PRRSV- or mock-infected cells were washed three times with phosphate-buffered saline (PBS), fixed with 4% paraformaldehyde for 15 minutes at room temperature, and then permeabilized with 0.2% Triton X-100 (Solarbio, Beijing, China) for 15 minutes. Following three washes with PBS, the cells were blocked with 3% bovine serum albumin (BSA, Solarbio, Beijing, China) for 1 hour at room temperature to minimize non-specific binding. Subsequently, the cells were incubated overnight at 4°C with an anti-PRRSV N protein primary antibody. After three additional washes with PBS to remove unbound primary antibodies, the cells were incubated with anti-mouse IgG secondary antibodies (Cell Signaling Technology, Danvers, MA, USA) for 45 minutes at room temperature. Representative images were captured and processed using an inverted fluorescence microscope for subsequent analysis.

### 2.4 Sequencing analysis

Sequence assembly and alignment were performed using Lasergene SeqMan and MegAlign (DNASTAR, Madison, WI, USA). Reference sequences retrieved from GenBank were aligned with the experimental data. For phylogenetic analysis, all trees were constructed using MEGA 7.0 software (https://www.megasoftware.net/).

### 2.5 Animal experiment design

To determine the pathogenicity of PRRSV ZJ01, we performed challenging studies on animals. Ten healthy 35-day-old piglets were selected and divided into two groups of five each. The challenge is performed by intramuscular injection of 2 ml of PRRSV ZJ01 culture containing 10^5^ TCID_50_, while the group serving as a negative control is injected with RPMI-1640. We recorded daily rectal temperature and clinical manifestations in both groups. Serum samples are taken weekly and analyzed for virus content and antibody levels. During the experiment, we tracked the survival status of the piglets. Eventually, on the 14th day (DPI) after the challenge, the piglets were euthanized.

The pigs used in this study were healthy, 30-day-old animals sourced from the Wens Group. All pigs were confirmed negative for major swine pathogens, especially PRRSV. Animal experiments were conducted and monitored within the standard animal facility at the Wens Research Institute.

### 2.6 Virus detection in animal experiments

Viral RNA load in samples from animal experiments was quantified using TaqMan real-time RT-qPCR. The assay employed the following primer and probe set specific for the PRRSV N gene: Forward primer: 5′-CTAGGCCGCAAGTACATYCTG-3′, Reverse primer: 5′-TTCTGCCACCCAACACGA-3′, Probe: 5′-FAM-TGATAACCACGCATTTGTCGTCCG-BHQ-3′ (dual-labeled). Thermal cycling conditions were: initial denaturation at 95°C for 20 seconds, followed by 40 cycles of denaturation at 95°C for 3 seconds and annealing/extension at 60°C for 30 seconds.

### 2.7 Serum antibody detection

PRRSV-specific antibody titers were measured using the HerdChek PRRSV X3 Antibody Test Kit (IDEXX Laboratories, Inc., Westbrook, ME) according to the manufacturer’s instructions. Antibody titers were reported as sample-to-positive (S/P) ratios. Serum samples with an S/P ratio ≥ 0.4 were considered positive.

### 2.8 Histopathology

At necropsy, lung tissues were fixed in 10% buffered neutral formalin and subsequently processed for hematoxylin and eosin (H&E) and immunohistochemical (IHC) staining using a Leica fully automated staining machine (Leica, Wetzlar, Germany), as previously described. Sections were examined under light microscopy at 200× magnification.

### 2.9 Statistical analysis

Each experimental group had its statistical significance evaluated through one-way analysis of variance (GraphPad Prism 9). Probability values were deemed statistically significant if they were less than 0.05 on both sides (P < 0.05).

## 3 Results

### 3.1 Virus isolation

Clinical samples were first screened for other swine pathogens, including porcine circovirus type 2 (PCV2), classical swine fever virus (CSFV), and pseudorabies virus (PRV). Samples testing positive solely for PRRSV were filtered and inoculated onto PAMs and observed cytopathic effect (CPE). Upon reaching approximately 90% CPE, both the cell culture supernatant and cells were harvested. After centrifugation, the clarified supernatant was passaged onto fresh PAM monolayers. Serial passage was continued in this manner until consistent CPE was observed. The identity of the virus in the resulting isolate was then confirmed by specific pathogen detection assays.

**Figure 1:**
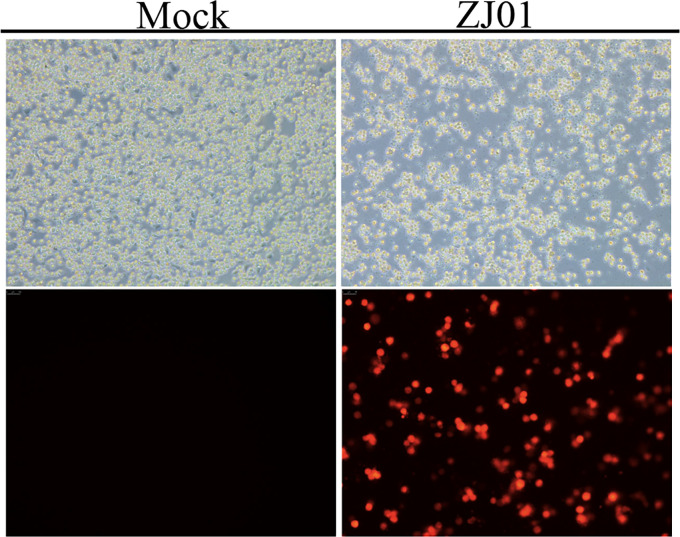
Strain isolation and identification.

To corroborate the presence of PRRSV, an indirect immunofluorescence assay (IFA) was conducted, and the specific PRRSV nucleocapsid (N) protein was detected within the infected cells, providing conclusive evidence of viral infection. Based on these findings, the isolated PRRSV strain was designated as ZJ01, marking a successful isolation of the virus (**Figure 1**).

### 3.2 Phylogenetic analysis

**Figure 2:**
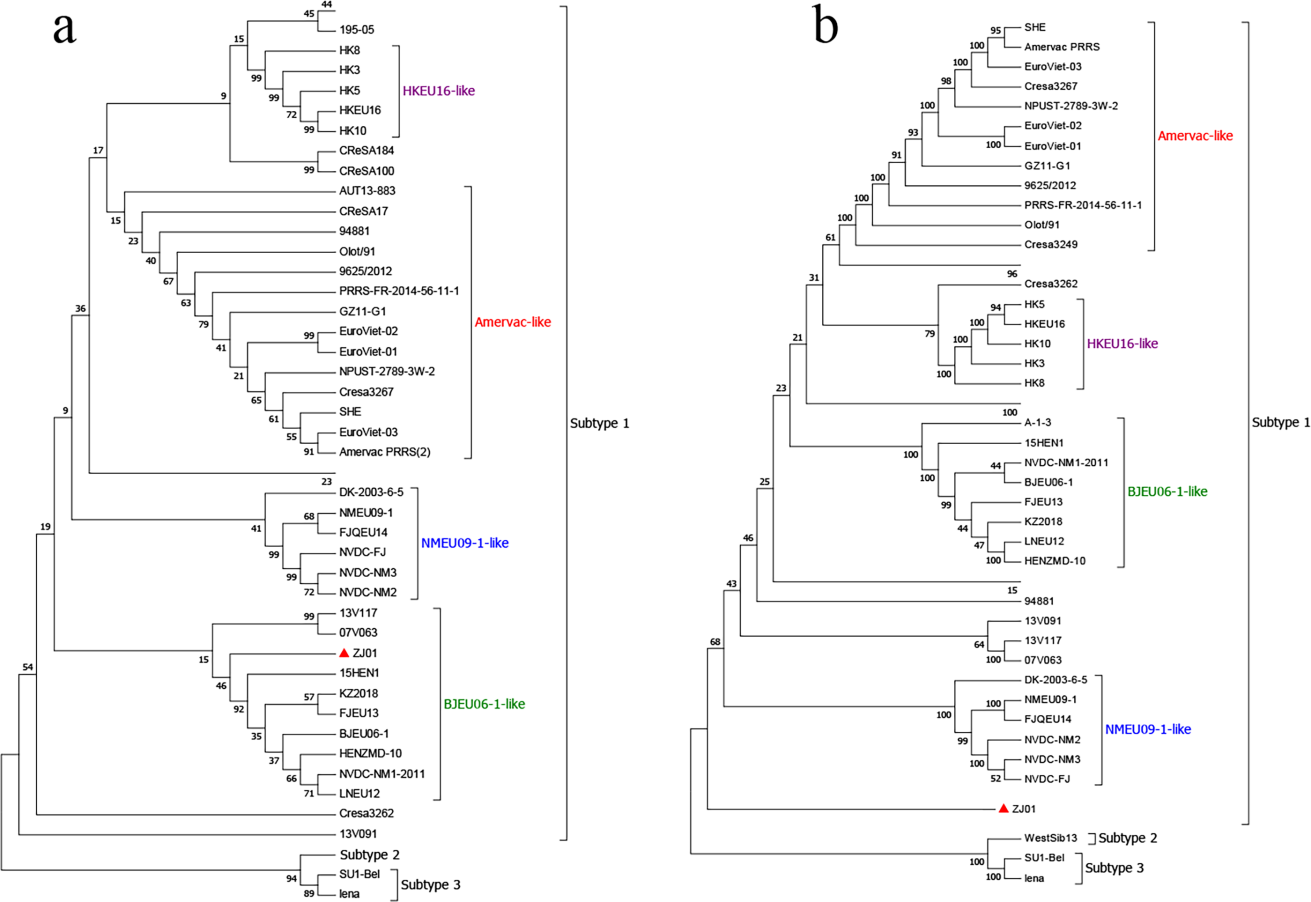
Phylogenetic analysis of ZJ01. **(a)** Phylogenetic trees constructed based on whole-length ZJ01genomes. **(b)** Phylogenetic trees constructed based on the GP5 gene of ZJ01.

The whole gene sequence of PRRSV ZJ01 strain was 15,125 bp long. The genetic evolution of the strain was further analyzed. The results showed that the whole genome sequence of PRRSV ZJ01 strain was classified as subtype 1, which did not belong to any clade of the current subtype, and was classified as a new clade (**Figure 2a**), and the homology of the existing subtype strains was low. Genetic and evolutionary analysis based on ORF5 showed that all PRRSV ZJ01 strains were classified as BJEU06–1 strain (**Figure 2b**).

### 3.3 GP3/4 amino acid analysis

**Figure 3:**
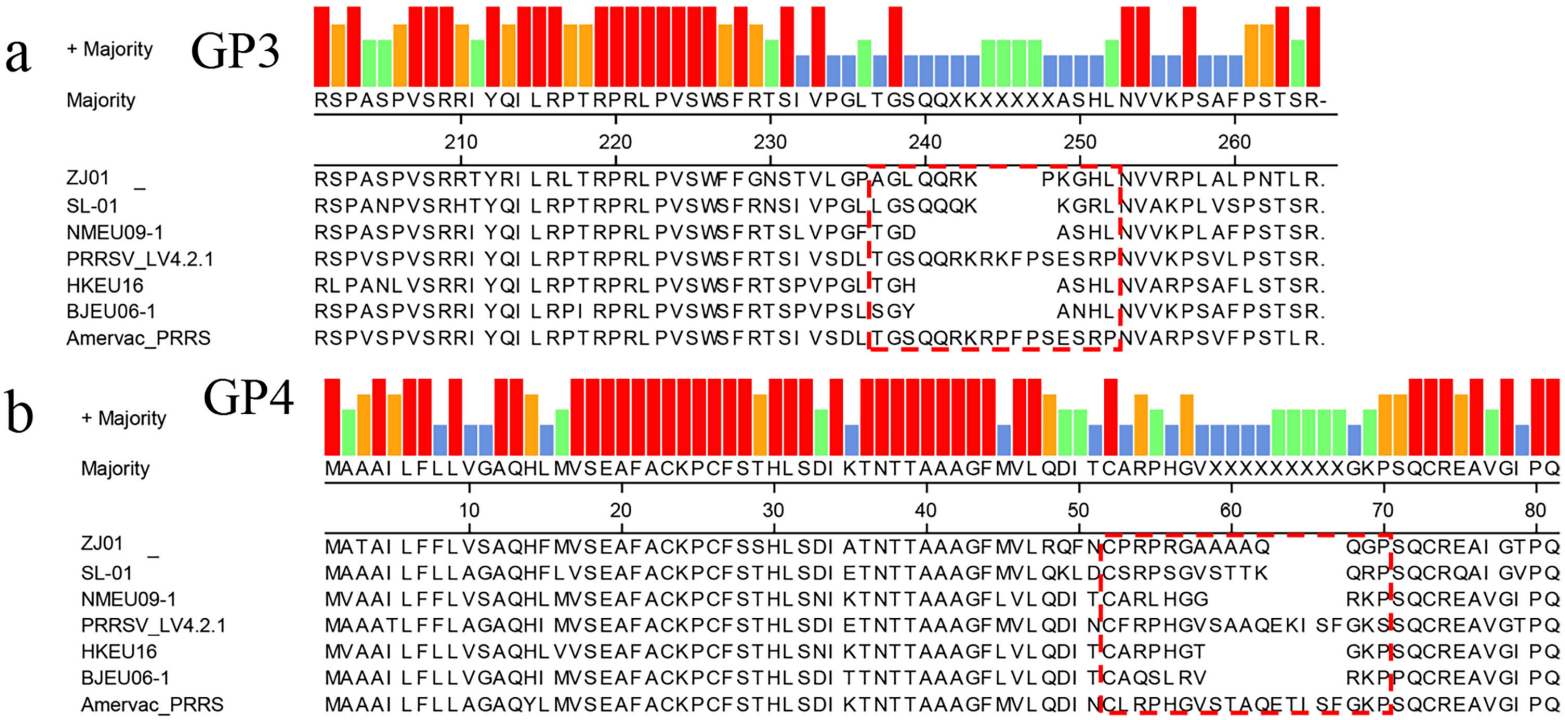
Amino acid sequence alignment based on GP3/4 amino acid. The red dotted line frame indicates the position of amino acid deletion.

Among the structural proteins of PRRSV-1, GP3 and GP4 possess hypervariable regions (GP3 aa 237–252; GP4 aa 57–72). Sequence analysis of these proteins in the ZJ01 strain revealed deletions within these regions: GP3 exhibited a three-residue deletion at positions 243–248 (**Figure 3a**), while GP4 contained a four-residue deletion at positions 63–68 (**Figure 3b**). Similar deletions have been reported in the NMEU09-1, HKEU16-1, and BJEU06–1 strains.

### 3.4 Pathogenic model study of piglets

**Figure 4:**
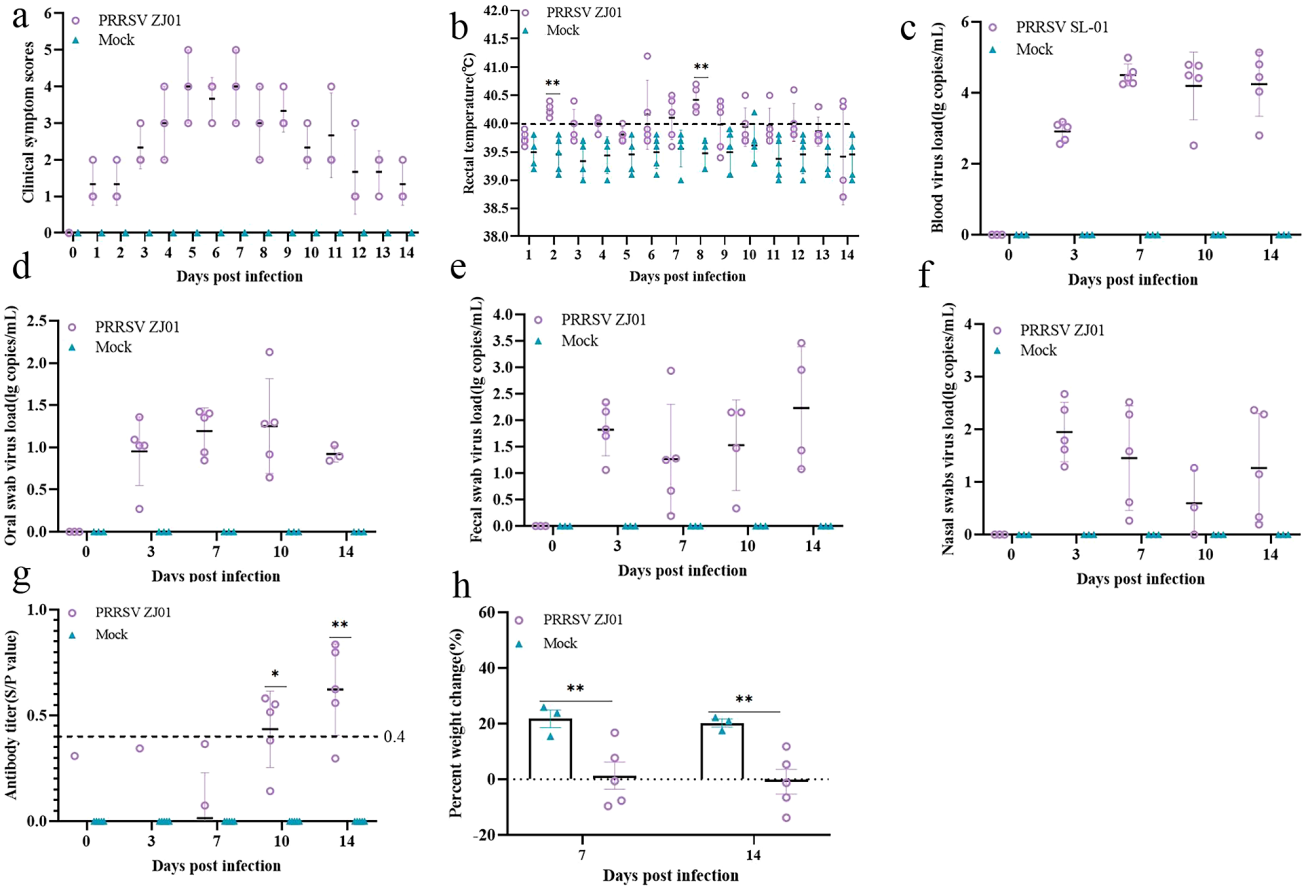
Animal pathogenic results. **(a)** Body temperature change of pigs in each group after challenge. **(b)** Clinical scores of pigs after challenge during the entire experiment. **(c-f)** Viral load detection in blood, oral swabs and pharyngeal swabs. **(g)** PRRSV‐specific antibody level was detected of each group during the challenge study. Each bar represents the average for all pigs in each group ± standard deviation (SD). The significant difference is marked with the asterisk, **P < 0.01, and *P < 0.05. **(h)** Body weight gains of each group during the challenge study.

The results of the experiment showed that the piglets began to have fever on the third and fourth days after infection, and the body temperature reached a peak on the sixth day, and then the body temperature gradually returned to normal levels (**Figure 4b**). During the whole experimental period, the body temperature of pigs infected with the ZJ01 strain was consistently higher than that of the uninfected control group, resulting in fever in pigs. In terms of clinical symptom scores, pigs infected with the ZJ01 strain reached the highest score on the seventh day, showing more obvious signs of onset than the control group (**Figure 4a**). In addition, the ZJ01 strain not only caused a high viral load in the blood, but also detected viral nucleic acid in oral and anal swabs, indicating that the strain can cause hyperviremia and excrete the virus through the mouth and feces (**Figures 4c–f**). Further testing of PRRSV N antibody in serum showed that pigs infected with the ZJ01 strain produced a positive reaction for PRRSV antibody and reached a high antibody level on the fourteenth day (**Figure 4g**). In addition, the weight gain of the challenged group was significantly lower than that of the blank group, indicating that the ZJ01 strain seriously affected the growth and fattening of pigs (**Figure 4h**).

### 3.5 Lung pathology observation

**Figure 5:**
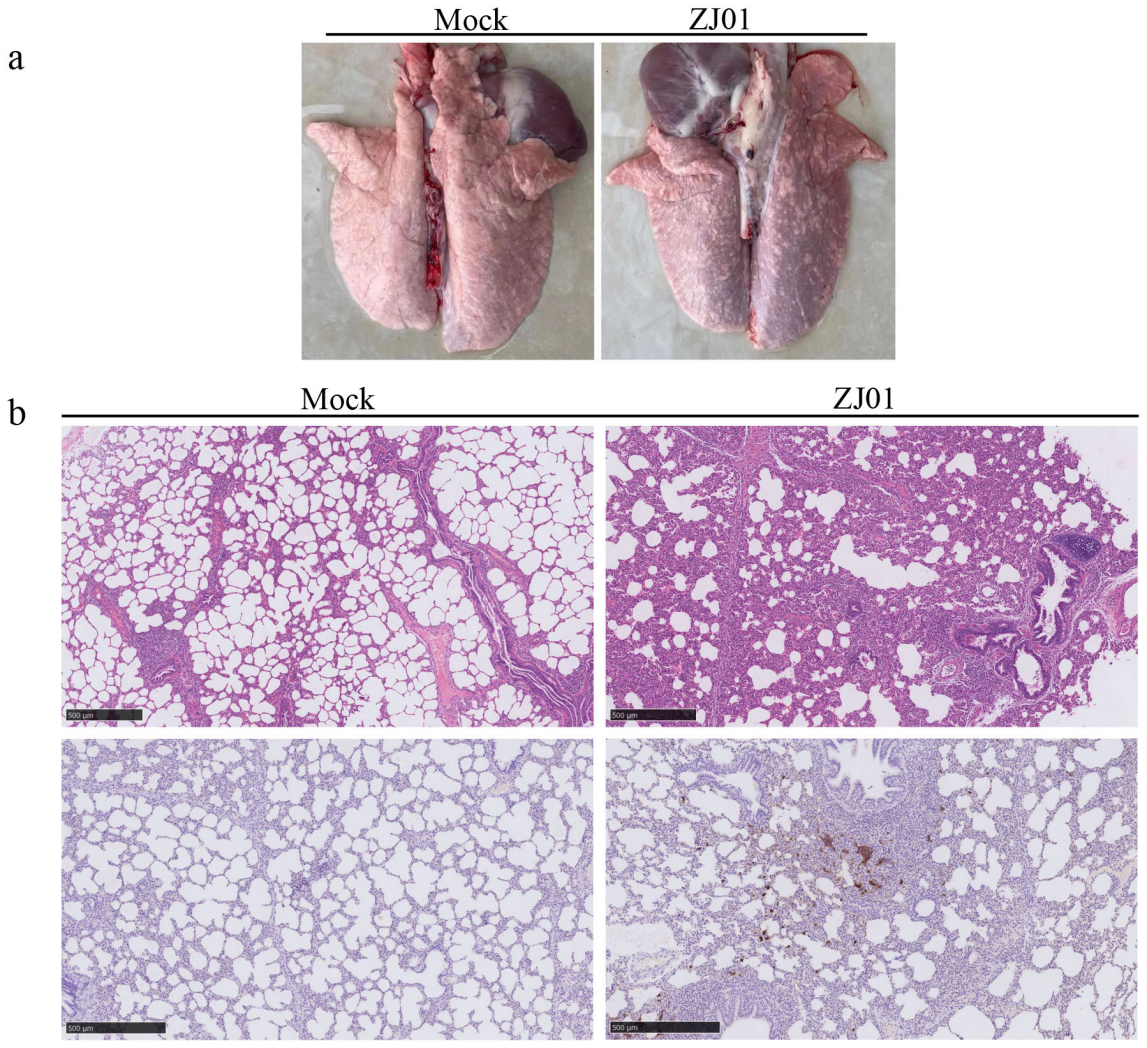
Observation and detection by pathological necropsy. **(a)** Necropsy observation of lung. White arrows mark the lesion site. **(b)** Histopathology tests of lung. Black arrows mark the lesion site.

During the experimental period, no mortalities were observed in any of the groups. On day 14 post-challenge, pigs in each group were euthanized. The lungs, submandibular lymph nodes, and inguinal lymph nodes were macroscopically examined and collected for further analysis. Necropsy findings showed that both viral strains induced localized pulmonary hemorrhage (**Figure 5a**). Subsequent histopathological evaluations revealed alveolar damage in infected groups, characterized by enlarged alveolar vacuoles, which were absent in the control group (**Figure 5b**).

## 4 Discussion

In 1997, PRRSV-1 infected pigs were first intercepted in China (Yuzhakov et al., 2017). In 2011, Wang X. et al. isolated the GZ11-G1 strain, which is highly homologous to the vaccine strain Amervac, which may be related to vaccinated breeding pigs (Sun et al., 2022). Since then, PRRSV-1 infections have been reported to increase in China, with a positive rate of 24.8 percent in 2016 for farms in Guangdong Province (Chen et al., 2011; Zhai et al., 2018; Li et al., 2023). After the outbreak of African swine fever, animal disease surveillance was strengthened, and the number of PRRSV-1 test reports increased. Currently, PRRSV-1 infection is endemic in at least 23 regions of China (Zhou et al., 2015; Wang et al., 2019; Wang et al., 2023).

The number of PRRSV-1 strains isolated from China has increased, but the pathogenicity is generally low (Canelli et al., 2017). Earlier reported strains such as HLJB11, GZ1-G2789 (Li et al., 2022), and NPUST-3-2W-34 belong to the Amervac-like subtype, while ZD-1291 and SD06 are similar to BJEU1-37. Strains such as GZ34-G1 and HLJB2789 exhibit moderate or low pathogenicity, but NPUST 2-1W-40 is less pathogenic (Chen et al., 2020). The same subtype of PR40/40/1291 isolated from Italy in 2014–1291 exhibits moderate pathogenicity (Zhao et al., 2021). The pathogenicity of SD79 and ZD-3 is relatively low, but the effect of premature C-terminal truncation of GP37 and GP3 on pathogenicity is unclear (Sun et al., 2023). Overall, the pathogenicity of PRRSV-1 strains in China has increased slightly, and highly pathogenic strains have been reported in neighboring countries and important importing countries (Wang et al., 2016; Chen et al., 2017; Zheng et al., 2024).

The overlapping ORF3 and ORF4 of PRRSV-1 are a hotspot for structural variation, often featuring deletions (Meulenberg et al., 1995; Lee et al., 2017). ORF3’s glycoprotein GP3 contains a carboxy-terminal hypervariable region (HVR, aa 237-252), which mutates rapidly under immune selection. Mutational hotspots at residues 57–72 and 237-252, including an 8-aa deletion (aa 241-248), are common in circulating strains (Meulenberg et al., 1997b). Our analysis of strain ZJ01 identified a 3-aa deletion in GP3’s HVR and a 4-aa deletion in GP4. These deletions, located within known hotspots, suggest coordinated evolution or selective pressures. Although distinct from the prevalent 8-aa deletion, ZJ01’s pattern highlights the genomic segment’s evolutionary plasticity.

Moreover, PRRSV can cause diseases such as fever death in piglets and reproductive disorders in pregnant sows, which has brought huge economic losses to the pig breeding industry. At present, PRRSV-1 has caused more clinical infections in Chinese pig herds, and the prevalence of new strains has brought great challenges to the immune control of pig farms. In this paper, a novel PRRSV1 ZJ01 strain was isolated, with a genome length of about 15,125 bp, and the results of genetic and evolutionary analysis showed that it belonged to a new subtype clade. The results of further piglet challenge test showed that, unlike the current reports, the latest ZJ01 strain had high pathogenicity to piglets, which could cause high body temperature and clinical symptoms, and the level of viremia was high. In this study, the biological characteristics and pathogenic model of the new PRRSV1 strain were studied, so as to provide reference and guidance for the prevention and control of pig breeding.

## 5 Conclusion

In this study, a novel PRRSV-1 ZJ01 strain was isolated and confirmed as a novel subtype by whole genome sequencing and genetic and evolutionary analysis. The ZJ01 strain can cause high fever and clinical symptoms in piglets with high levels of viremia. This study provides a new reference and guidance for the prevention and control of pig breeding.

## Data Availability

The datasets presented in this study can be found in online repositories. The names of the repository/repositories and accession number(s) can be found in the article/supplementary material.
